# Dilation of the cystic duct confluence in laparoscopic common bile duct exploration and stone extraction in patients with secondary choledocholithiasis

**DOI:** 10.1186/s12893-020-00705-y

**Published:** 2020-03-17

**Authors:** Xiao-Bin Yang, An-Shu Xu, Jian-Gang Li, Yong-Ping Xu, De-Song Xu, Chao-Chun Fu, Da-Bo Deng, Jie Li, Ma-Zhong Zhang

**Affiliations:** 1 Department of General Surgery, The First People’s Hospital, No. 1 Yuanlin Road, Qujing City, 655000 Yunnan Province China; 2 Department of Anesthesiology, The First People’s Hospital, No. 1 Yuanlin Road, Qujing City, 655000 Yunnan Province China; 3 Department of Anesthesiology, Shanghai Children’s Medical Center, Shanghai Jiao Tong University School of Medicine, Shanghai, China

**Keywords:** Cystic duct, Cholelithiasis, Secondary choledocholithiasis, Common bile duct exploration, Safety, Efficiency

## Abstract

**Background:**

Many options exist for the management of cholelithiasis and secondary choledocholithiasis. Among them, laparoscopic common bile duct exploration (LCBDE) with choledocotomy followed by laparoscopic cholecystectomy has gained popularity. However, efforts should be made to ensure minimally invasive or noninvasive management of the common bile duct (CBD). The purpose of this study was to explore the clinical experience of non-invasive surgical modality, i.e., laparoscopic transcystic dilation of the cystic duct confluence in CBD exploration (LTD-CBDE), including feasibility, safety, adverse events, and incidence.

**Methods:**

In this retrospective analysis, 68 patients were offered the LTD-CBDE technique from December 2015 to April 2018 based on patient’s own intention. During the surgery, the cystic duct confluence was dilated with separation forceps and/or a columnar dilation balloon. Subsequently, CBD exploration and stone extraction were performed with a choledochoscope. The entrance of the CBD was covered with a cystic duct stump wall and was subjected to primary closure at the end of surgery.

**Results:**

Forty-nine females and 19 males with cholelithiasis and secondary choledocholithiasis were included. The mean age was 53 years old (18 to 72 year). Of these patients, 62 (91.2%) were successfully treated with the LTD-CBDE technique, and bile leakage was observed in 3 patients (4.4%). The mean operation time was 106 min, and the mean hospital stay was 5.9 days. Among the other 6 patients, 3 were converted to open cholecystectomy due to severe fibrosis, unclear anatomical structure at Calot’s triangle (*n* = 2) or Mirizze syndrome (*n* = 1); LCBDE was performed in 3 patients due to cystic duct atresia (n = 2) and low level of flow from the gallbladder duct into the CBD (*n* = 1). These patients had a smooth postoperative course. In total, 43/68 of the patients presented no radiological evidence of retained CBD stones at the postoperative follow-up (40 patients treated with LTD-CBDE) 1 year later.

**Conclusions:**

The current work suggests that LTD-CBDE for the management of cholelithiasis and secondary choledocholithiasis is a feasible, safe and effective technique with a low complication rate. LTD-CBDE offers another alternative for surgeons to treat patients in similar scenarios. However, additional randomized, controlled studies are needed to demonstrate its efficacy, safety, and impact on CBD stenosis.

## Background

The incidence of common bile duct (CBD) stones reported in the literature in patients with gall bladder stones varies between 7 and 20% [1–3]. Shortly after Bobbs performed the first cholecystectomy in 1867, Abbe performed the first CBD exploration [4]. Subsequently, open cholecystectomy and CBD exploration followed by T-tube drainage gradually became a classic surgical modality for patients with cholelithiasis and secondary choledocholithiasis. With the introduction of endoscopy and laparoscopy into the clinic in the 1970s and 1980s, accompanied by the evolution of widespread expertise, laparoscopic cholecystectomy (LC) has been rapidly accepted as a routine treatment for patients with symptomatic gall bladder stones and has gradually become the conventional choice for the management of CBD stones [5]. In China, Yunnan Province is an area with a high prevalence of lithiasis. A hospital in Qujing City first introduced laparoscopic techniques to China in 1991, and more than 100 LCs were performed that year.

Several clinical management methods are available for patients with cholelithiasis and secondary choledocholithiasis. Although the ideal method is still debated, endoscopic retrograde cholangiopancreatography (ERCP, two-stage) and laparoscopic common bile duct exploration (LCBDE) with choledocotomy followed by LC (LCBDE, single-stage) are two commonly used methods [5–8]. Compared with ERCP followed by LC, LCBDE has the advantage of a higher success rate and a shorter hospital stay while simplifying the two procedures into a single minimally invasive operation [5]. However, in most cases, the LCBDE modality has to be performed via a choledochotomy followed by T-tube drainage. T-tube placement presents difficulties in postoperative management [9]. Thus, Chen et al. [10] and Niu et al. [11] modified the transcystic approach with a microincision of the cystic duct confluence or CBD followed by a primary suture without a T-tube. The results revealed good preliminary effects. However, we think that a microincision is still a kind of injury to the CBD, and in the long run, patients might suffer from CBD stenosis, especially patients who had CBD stenosis before the operation.

**Fig. 1:**
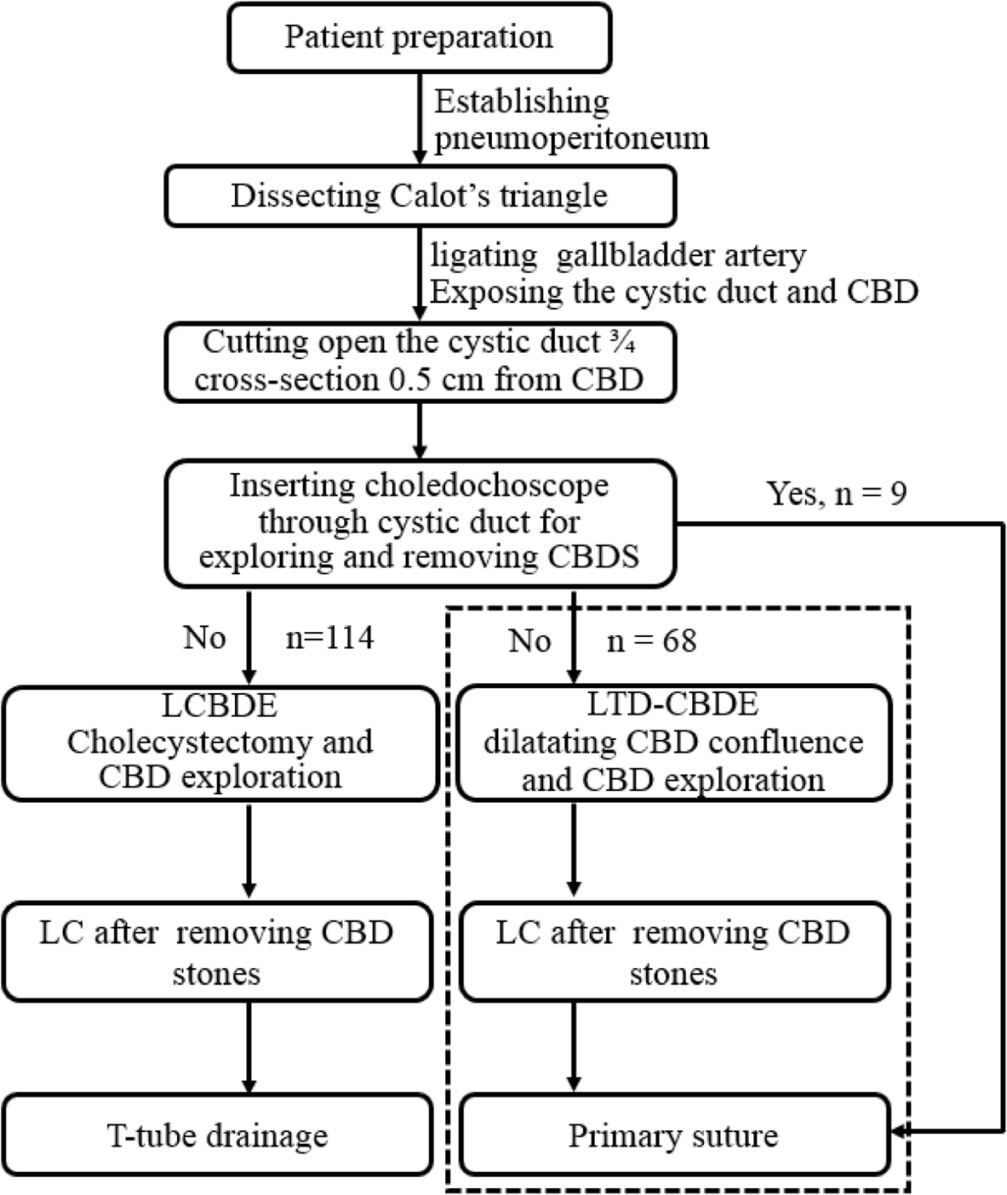
Detailed surgery flow diagram. Among 191 patients with cholelithiasis and secondary choledocholithiasis, CBD stones were successfully extracted through the cystic duct and confluence with the choledochoscope in 9 patients, while 68 patients received LTD-CBDE (dotted box) and 114 patients received LCBDE. CBD, common bile duct; LCBDE: laparoscopic CBD exploration with choledocotomy followed by laparoscopic cholecystectomy; LTD-CBDE: laparoscopic transcystic approach by dilating the cystic duct confluence in CBD exploration. LC: laparoscopic cholecystectomy

Therefore, efforts should be made to minimize injury to the cystic duct confluence or CBD, preferably resulting in “no injury”. For this purpose, we modified the surgical modality by replacing choledochotomy or confluence incision with laparoscopic transcystic dilation of the cystic duct confluence in CBD exploration (LTD-CBDE). Two issues, namely the feasibility and the patient safety of this new surgical procedure, should be raised and elaborated first before reviewing our clinical experience: (1) Feasibility of the surgical procedure. It is well known that secondary CBD stones originate from gallbladder stones [12]. They will pass through the gallbladder, cystic duct and the cystic duct confluence successively, and eventually fall into the CBD, which indicating that it is theoretically feasible to extract stones through the cystic duct confluence. The improved procedure takes full advantage of the natural tube of the cystic duct and avoids CBD incision. (2) Patient safety. If choledochoscope can be successfully inserted into the CBD without dilating the confluence, non-invasive stones extraction will be the standard surgical procedure. In this study, standard surgical procedures (Fig. 1) were performed in 9 patients. Otherwise, LCBDE or LTD-CBDE can be chosen. LCBDE is an invasive operation requiring a CBD incision. The “new” of LTD-CBDE regards to the dilation of the confluence. If it successes, non-invasive stones extraction can be performed without traditional CBD incision or micro-incision; if it fails, the CBD incision or micro-incision will have to be chosen [5, 10, 11] – the operation will be transformed into LCBDE technique again. In general, the current surgical procedure is to reduce rather than increase the injury to the patient with the ultimate goal of improving the patient safety and outcome. However, the patient safety guidelines and the ethics paradigm should be established before a new surgical technique being used, so we consulted the institutional review board (IRB) office and obtained the permission of performing the “new” surgical procedure. In addition, the current study was a retrospective analysis, as the original purpose was to provide the patients with a relatively non-invasive surgical approach rather than test a hypothesis. Although a retrospective review is usually discouraged, it can offer excellent strength and validity, and represents a valuable type of research [13]. Retrospective study was appropriate in the case of a small number of cases, help to clarify the hypothesis and identify feasibility issues for a prospective study [14]. This study retrospectively analyzed the clinical data of LTD-CBDE patients, with an emphasis on assessing the feasibility, safety, and effectiveness based on our preliminary experience of the LTD-CBDE technique.

## Methods

### Patients

The new surgical modality was reviewed and approved by the IRB at The Qujing First People’s Hospital. All patients were informed about the detailed surgical procedures of LCBDE and LTD-CBDE, the surgical modality was decided according to patient’s intention and written informed consent was signed. The IRB waived the requirement for patient informed consent given the retrospective nature of the current work. From Dec. 2015 to Apr. 2018, 191 patients were diagnosed with cholelithiasis and secondary choledocholithiasis by preoperative ultrasound (US) and/or magnetic resonance cholangiopancreatography (MRCP) examinations and refused ERCP. In 9 patients, CBD stones were successfully extracted through the cystic duct and confluence with the choledochoscope, while 68 patients were offered LTD-CBDE, and the other 114 patients underwent classic single-stage LCBDE. Additionally, the outer diameter, location and size of the CBD stones were clearly defined and recorded. The patients who presented with acute cholangitis and acute gallbladder pancreatitis were excluded from receiving LTD-CBDE. All patients underwent routine preoperative examination, including chest X-ray, electrocardiogram, a routine blood test, liver and kidney function tests, and coagulation function tests.

### Surgical technique

**Fig. 2:**
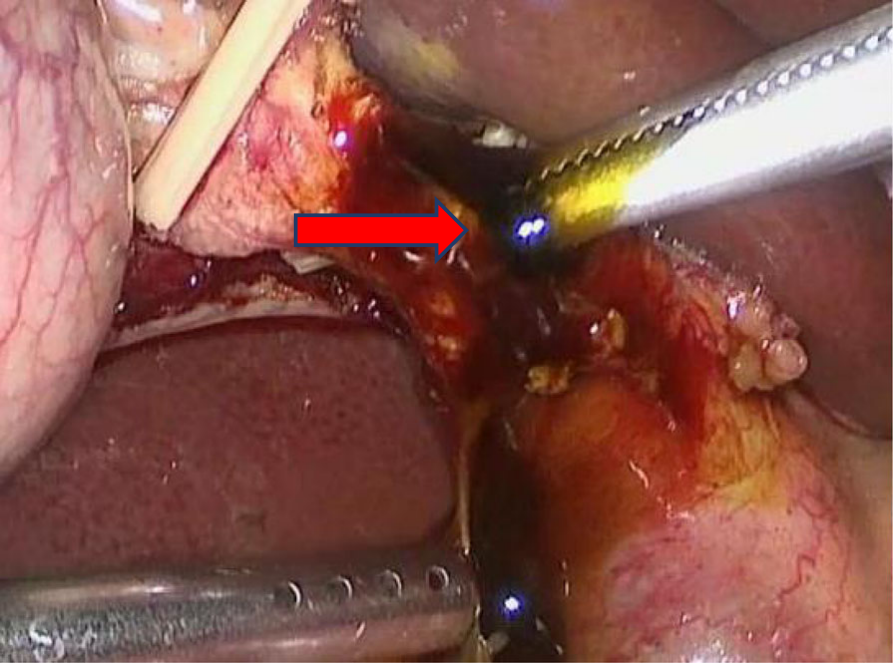
The confluence was dilated with the separation forceps (red arrow)

**Fig. 3:**
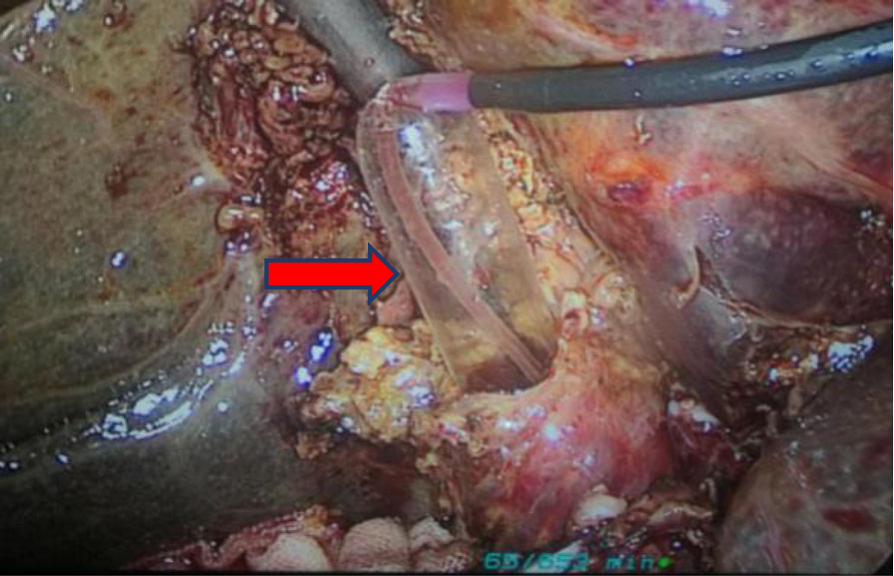
The confluence was dilated with the columnar dilation balloon (red arrow)

**Fig. 4:**
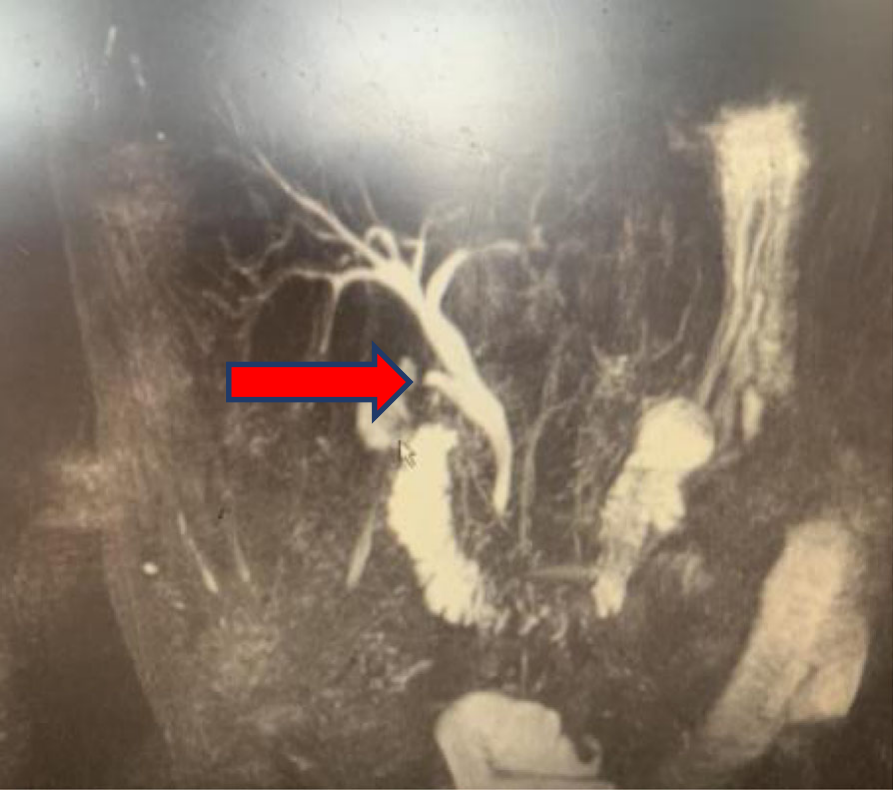
The stump wall of the cystic duct covered the entrance of the CBD following primary closure. Postoperative magnetic resonance cholangiopancreatography (MRCP) examination shows good healing (red arrow)



Operations were performed by the same surgical team with the patient under general anesthesia and with endotracheal intubation. During the operation, the patients were placed in reverse Trendelenburg positions, tilted to the left. Pneumoperitoneum was established with carbon dioxide at a pressure of 12–15 mmHg, which was adjusted as needed. Four trocars were used for LC according to the standard technique. The detailed surgery flow diagram is shown in Fig. 1. Calot’s triangle was dissected, and the cystic artery, cystic duct and CBD were exposed. The cystic artery was clipped and ligated first. Then, the cystic duct was clipped very near the gall bladder, and 3/4 of its cross section was cut open approximately 0.5 cm from the CBD. The choledochoscope (4.9 mm, Olympus, Tokyo, Japan) was inserted through the cystic duct and confluence for CBD exploration and stone extraction (success in 9 patients). Stones were removed with a Cook basket (NTSE-045065-UDH). If the removal failed, one of the following steps was performed: (i) LCBDE was performed (*n* = 114); (ii) the ventral side of the cystic duct was longitudinally incised 0.2–0.3 cm from the abovementioned incision of the cross section, and the confluence was dilated through the cystic duct with the separation forceps (Fig. 2), and then the choledochoscope was inserted again; or (iii) a columnar dilation balloon (M00558410) was used to dilate the confluence (Fig. 3) until the choledochoscope was successfully inserted followed by CBD exploration and stone extraction. The choledochoscope was also turned upward to explore the common hepatic duct and intrahepatic ducts. If the intrahepatic ducts presented no stones, then the distal CBD was explored again. Intraoperatively, the 68 patients reported herein (red dotted box in Fig. 1) were selected randomly to receive the surgical methods (LTD-CBDE modality) described in (ii) and (iii) above. As far as our practical experience was concerned, the dilation degree of the cystic duct and/or the confluence depended on the size of the largest stone, which was determined based on preoperative US or MRCP examination. After the stones were completely removed (the CBD was completely clear and no evidence of remnant stones), the cystic duct was cut, and LC was performed. The stump wall of the cystic duct was used to cover the entrance of the CBD, primarily by laparoscopic-interrupted full-thickness suturing, as shown in Fig. 4. The abdominal drainage tube was left in the small omentum hole in all patients. Those interested can refer to the full video shown in the supplementary material.

**Additional file 1**



### Outcomes assessment

The patients’ demographic data and the following indicators were collected and recorded: operation time in minutes, surgical success rate, open conversion rate, residual stone rate, timing of postoperative return of peristaltic activity and food intake, postoperative hospital stay, and postoperative bile leakage rate. Before the patients were discharged from the hospital, they were instructed to pay attention to the clinical symptoms, signs, and laboratory and imaging findings related to possible biliary diseases after surgery. A follow-up MRCP evaluation was performed 1 year after surgery.

### Statistical analysis

Descriptive statistics were performed with Excel. Data are presented as numbers, percentages, and arithmetic means ± standard deviations (SDs) where appropriate.

## Results

Sixty-eight patients with cholelithiasis and secondary choledocholithiasis were randomly offered LTD-CBDE. Table 1 shows the patient demographics and characteristics of lithiasis. The patient population comprised 19 (27.9%) males and 49 (73.1%) females, with a mean age of 53 ± 14 years old (ranging between 18 and 72 yr). Among the patients, 52 presented with symptoms and signs of obstructive jaundice, and 8 patients had a previous abdominal surgery history. The presence of gallbladder stones was confirmed by US examinations, and the CBD stones were initially diagnosed by US and confirmed again by MRCP in all patients. The mean measured outer diameter of the CBD was 12.6 ± 1.8 mm (ranging from 9 to 17 mm). The median largest diameter of the CBD stone was 9 mm (ranging from 0.3 to 1.1 cm).

**Table 1:** Patient demographics and characteristics of lithiasis (*n* = 68)

Variable	Value
Sex	
Men	19 (27.9%)
Women	49 (72.1%)
Mean age, year (range)	53 ± 14 (18–72)
Previous abdominal surgery history	8 (11.8%)
ASA I/II/III	42/20/6
Number of CBD stone	
1	54
2–3	12
> 3	2
Median diameter of largest stone, mm (range)	9 (3–11)

Values are expressed as the mean ± SD, median or number of patients (%)

*ASA* American Society of Anesthesiologists physical status, *CBD* common bile duct

Of the 68 patients, 62 (91.2%) underwent LTD-CBDE successfully, and the CBD was dilated with columnar dilation balloons (in 5 patients) and separation forceps (in 57 patients). The mean operation time was 106 min (ranging from 90 to 120 min). Patients with ASA III grade stayed in the ICU for 1–4 days, and the rest of the patients returned to the ward. All patients regained peristaltic activity on the next postoperative day and consumed liquid food the next early morning. The mean postoperative hospital stay was 5.9 days (4–15 days). The outcomes related to the modified LTD-CBDE modality are shown in Table 2.

**Table 2:** The outcomes related to modified surgical modality (*n* = 62)

Variable	Value
Mean operation time, minutes (range)	106 ± 9 (90–120)
Success rate	62/68 (91.2%)
Common bile stone clearance rate	62 (100%)
Retained stones with postoperative MRCP	0 (0%)
Mean postoperative hospital stay, days (range)	5.9 ± 2.4 (4–15)

Values are expressed as the mean ± SD or number of patients (%)

*MRCP* magnetic resonance cholangiopancreatography

As shown in Table 3, among the 62 patients who successfully received LTD-CBDE, bile leakage was observed in 3 patients (4.4%). The maximum bile drainage volume in these 3 patients was less than 100 mL/day, and the bile leakage stopped within 2 days. The abdominal drains were removed within 24–48 h postoperatively. Among the 62 patients, there was no evidence of residual stones or CBD stenosis with routine MRCP examinations before discharge. Forty patients returned to the hospital for MRCP re-examination 1 year after surgery, and none showed signs of CBD recurrence or stenosis.

**Table 3:** Complications related to the modified surgical modalities and treatments of failure cases

Variable	Value
Complications	
Pancreatitis	0/62(0%)
Biliary leakage	3/62 (4.8%)
Conversion open rate	3/68 (4.4%)
Conversion choledochotomy + T-tube	3/68 (4.4%)
Number of patients with follow-up 1 year later	43/68 (63.2%)

Values are expressed as the number of patients (%)

Of the 68 patients included, 6 (8.8%) were treated by other surgical methods for various reasons. In 2 patients with fibrosis and unclear anatomical structures at Calot’s triangle and 1 patient with coexisting Mirizze syndrome, the procedure was converted to open cholecystectomy, and the CBD was explored to remove the stones safely with T-tube drainage. In 2 patients with cystic duct atresia and in 1 patient with low confluence opening, LCBDE and T-tube drainage were performed. Postoperative MRCP revealed patency of the CBD. These six patients had a smooth postoperative course. Three patients returned to the hospital for MRCP re-examination 1 year after surgery, and none showed signs of CBD recurrence or stenosis.

## Discussion

Secondary CBD stones may cause many clinical symptoms and signs, including abdominal pain, obstructive jaundice, cholangitis, and biliary pancreatitis [2]. Among the patients in this article, there were as many as 52 patients diagnosed with obstructive jaundice. The ideal management of this condition remains a matter of debate [5]. As far as LCBDE is concerned, the problems are mainly related to T-tube placement, such as patient discomfort, biliary peritonitis, and T-tube displacement [15, 16]. In view of this, some surgeons tried to make full use of the natural cystic duct with a microincision of the CBD or confluence followed by primary suture [10, 11]. Inspired by this, we further speculate: could we use the natural orifices comprising the cystic duct and its confluence at the CBD with no incision? Therefore, LTD-CBDE was designed for CBD exploration and stone extraction via a laparoscopic transcystic approach by dilating the confluence. Dilation of the confluence makes the insertion of the choledochoscope and stone extraction easier because it not only overcomes the problems that the cystic duct is thin and the spiral valve acts as a barrier during exploration but also enlarges the inlet diameter of the CBD to be greater than or equal to the outer diameter of the largest stone. CBD blood supply is not affected by the incision, so CBD stenosis may be prevented. The operations were performed smoothly. The success rate was 91.2% (which may be higher with careful perioperative identification of the indications), the clearance rate of CBDS was 100%, and retained stones were not identified at the postoperative follow-up.

The success rate of 91.2% with the present method coincides with that of 88.1% reported in a meta-analysis of 11 randomized trials of LCBDE [5]. By using a slightly different approach with a microincision at the confluence, Chen et al. and Niu et al. reported a success rate of 100% [10, 11]. The 6 failed patients in our work were associated with anatomical problems, suggesting the importance of a carefully selected surgical strategy. Special attention should be paid to the following aspects. First, it is crucial to maintain the cystic duct intact to facilitate the incision, dilation, choledochoscope insertion, observation, stone extraction and primary closure. Second, it is important to identify the confluence correctly and avoid its damage. Finally, factors limiting the success of LTD-CBDE include anatomic features related to the cystic duct and confluence, such as fibrosis and anatomical abnormality of Calot’s triangle; small-size, atretic or tortuous duct; and low level of or posterior insertion of the cystic duct on the CBD.

The operation time reported in the literature varies widely depending on the surgical method, ranging from 104 to 194 min [2, 10, 17, 18]. The mean operating time was 105 min in our series. However, we do not think it is reasonable to directly compare the operation time because any new modality requires more operation time and is technically difficult with a clear learning curve. In the future, along with technical improvement and more effective logistic organization, the operation time will be further reduced. Patients in our series were discharged after a mean postoperative hospital stay of 5.9 days, which is not longer than other reports of mini-incision (mean 8 days) or LCBDE [5, 17, 18]. Mortality was also in accordance with the findings of other surgical modalities [10, 11]. Forty-three patients were followed up 1 year after the LTD-CBDE operation (25 patients lost to follow-up), and none of them presented with evidence of retained or recurrent CBDS or stenosis of CBD.

Our LTD-CBDE method is safe and effective, but a carefully selected surgical strategy should be especially emphasized, as suggested by Gigot et al [18] First, for patients with anatomical abnormalities or intraperitoneal adhesions, as shown in 6 patients in our series, the traditional open operation or laparoscopic choledochotomy should be performed as soon as possible. Second, despite careful suturing of the confluence with the stump wall, there were still 3 patients who suffered postoperative bile leakage. This is not higher than the incidence associated with LCBDE (5.6% with experienced surgeons vs 17.1% with inexperienced surgeons) reported by Liu et al [19] As analyzed by Liu et al. [19], it is clear that postoperative bile leakage (and the like) can be reduced by gaining experience in the technique [20]. Third, in our series, separation forceps, rather than balloons, were used in most patients. It is undeniable that the latter provides a quantitative and accurate degree of expansion, thus improving safety accordingly. However, Yunnan is a poor province in China, and most patients cannot afford the expensive balloon. Therefore, we chose separation forceps rather than balloons to dilate the confluence, and fortunately, the majority of patients for whom separation forceps were used to dilate the confluence successfully underwent surgery with the LTD-CBDE technique. Fourth, this is a retrospective analysis. The surgical method was selected subjectively rather than randomly, which is why we did not used the remaining 114 patients with LCBDE as a control group. Robust RCT research will be our next goal. Finally, although we hypothesized that LTD-CBDE has the potential to reduce postoperative bile duct stricture, this has not been confirmed, and further research is needed. The optimal management of CBDS depends on the skills and techniques of the surgical team available. In any case, minimally invasive or noninvasive procedures should be the direction of our efforts.

## Conclusion

We modified the surgical modality by dilating the cystic duct confluence in CBD exploration (LTD-CBDE). LTD-CBDE is a safe and effective surgical procedure for patients with secondary choledocholithiasis, although it requires clinical experience as well as advanced laparoscopic skills. However, additional randomized, controlled studies are needed to demonstrate its efficacy, safety, and impact on CBD stenosis, especially in patients with preoperative CBD stricture.

## Data Availability

The data used and materials related to the current work are available from the corresponding author (ASX) or the first author (XBY) on reasonable request.

## Abbreviations

CBD: Common bile duct
ERCP: Endoscopic retrograde cholangiopancreatography
LC: Laparoscopic cholecystectomy
LCBDE: Laparoscopic common bile duct exploration with choledocotomy followed by laparoscopic cholecystectomy
LTD-CBDE: Laparoscopic transcystic approach by dilating the cystic duct confluence in CBD exploration
MRCP: Magnetic resonance cholangiopancreatography
US: Ultrasound

## References

[1] Neuhaus H, Feussner H, Ungeheuer A, Hoffmann W, Siewert JR, Classen M (1992). Prospective evaluation of the use of endoscopic retrograde cholangiography prior to laparoscopic cholecystectomy. Endoscopy.

[2] Ghazal AH, Sorour MA, El-Riwini M, El-Bahrawy H (2009). Single-step treatment of gall bladder and bile duct stones: a combined endoscopic–laparoscopic technique. Int J Surg.

[3] Sarli L, Pietra N, Franze A, Colla G, Costi R, Gobbi S, Trivelli M (1999). Routine intravenous cholangiography, selective endoscopic retrograde cholangiography and endoscopic treatment of common bile duct stones before laparoscopic cholecystectomy. Gastrointest Endosc.

[4] Ellis PH (2019). The story of gallstones and their treatment. J Perioper Pract.

[5] Singh AN, Kilambi R (2018). Single-stage laparoscopic common bile duct exploration and cholecystectomy versus two-stage endoscopic stone extraction followed by laparoscopic cholecystectomy for patients with gallbladder stones with common bile duct stones: systematic review and meta-analysis of randomized trials with trial sequential analysis. Surg Endosc.

[6] Andriulli A, Leandro G, Niro G, Mangia A, Festa V, Gambassi G, Villani MR, Facciorusso D, Conoscitore P, Spirito F, De Maio G (2000). Pharmacologic treatment can prevent pancreatic injury after ERCP: a meta-analysis. Gastrointest Endosc.

[7] Dickinson RJ, Davies S (1998). Post-ERCP pancreatitis and hyperamylasaemia: the role of operative and patient factors. Eur J Gastroenterol Hepatol.

[8] Nzenza TC, AI-Habbal Y, Guerra GR, Manolas S, McQuillan T (2018). Recurrent common bile duct stones as a late complication of endoscopic sphincterotomy. BMC Gastroenterol.

[9] Podda M, Polignano FM, Luhmann A, Wilson MS, Kulli C, Tait IS (2016). Systematic review with meta-analysis of studies comparing primary duct closure and T-tube drainage after laparoscopic common bile duct exploration for choledocholithiasis. Surg Endosc.

[10] Chen XM, Zhang Y, Cai HH, Sun DL, Liu SY, Duan YF, Yang C, Jiang Y, Wu HR (2013). Transcystic approach with micro-incision of the cystic duct and its confluence part in laparoscopic common bile duct exploration. J Laparoendosc Adv Surg Tech A.

[11] Niu X, Song J, He X, Chen J, Xu J, Li Z, Long H, Wei J (2018). Micro-incision of the cystic duct confluence in laparoscopic common bile duct exploration for elderly patients with choledocholithiasis. Indian J Surg.

[12] Chung EJ, Kim MH, Lee SS, Lee SK (2003). Primary vs. secondary common bile duct stones: apples and oranges. Endoscopy.

[13] Perry JD, Parrish RK, Goodman KW. The Prospective retrospective study. Am J Ophthalmol 2018;196:xiii-xv. 10.1016/j.ajo.2018.09.006 30376956

[14] Hess DR (2004). Retrospective studies and chart reviews. Respir Care.

[15] Decker G, Borie F, Millat B (2003). One hundred laparoscopic choledochotomies with primary closure of the common bile duct. Surg Endosc.

[16] Hiromi T, Akiko U, Hui C (2002). Laparoscopic management of common bile duct stones: transcystic approach and choledochotomy. J Hepato-Biliary-Pancreat Surg.

[17] Darkahi B, Liljeholm H, Sandblom G (2016). Laparoscopic common bile duct exploration: 9 year experience from a single center. Front Surg.

[18] Gigot JF, Navez B, Etienne J, Cambier E, Jadoul P, Guiot P, Kestens PJ (1997). A stratified intraoperative surgical strategy is mandatory during laparoscopic common bile duct exploration for common bile duct stones. Lessons and limits from an initial experience of 92 patients. Surg Endosc.

[19] Liu D, Cao F, Liu J, Xu D, Wang Y, Li F (2017). Risk factors for bile leakage after primary closure following laparoscopic common bile duct exploration: a retrospective cohort study. BMC Surg.

[20] Zhu H, Wu L, Yuan R, Wang Y, Liao W, Lei J, Shao J (2018). Learning curve for performing choledochotomy bile duct exploration with primary closure after laparoscopic cholecystectomy. Surg Endosc.

